# Effectiveness and safety of different traditional Chinese medicine therapies for allergic rhinitis: A protocol for systematic review and network meta analysis

**DOI:** 10.1097/MD.0000000000022521

**Published:** 2020-10-23

**Authors:** Ting Yu, ShiFan Yan, ZhenHai Chi, Pan Cheng, HaiYan Li, SiYu Qin, MingHui Li, RiXin Chen, Lin Jiao

**Affiliations:** a Jiangxi University of Traditional Chinese Medicine; b Affiliated Hospital of Jiangxi University of Traditional Chinese Medicine, Nanchang, China.

**Keywords:** allergic rhinitis, network meta-analysis, protocol

## Abstract

**Introduction:**

Traditional Chinese medicine has been widely used in the treatment of allergic rhinitis. However, currently randomized controlled trials (RCTs) and meta-analysis only compare 1 or 2 types of traditional Chinese medicine therapies, and the comprehensive ranking of efficacy and safety of multiple traditional Chinese medicine therapies for the treatment of allergic rhinitis has not been completed. Therefore, the purpose of this network meta-analysis is to evaluate the efficacy and safety of different traditional Chinese medicine therapies for the treatment of allergic rhinitis.

**Methods and analysis:**

Three English databases of PubMed, Embase, Cochrane Library, and 2 Chinese databases of CNKI and Wanfang were searched from their inceptions to September 1, 2020. At the same time, in order to prevent omissions, we also compared the previous meta-analysis to determine the final included trials. The main evaluation outcome was the total Clinical Score (total nasal symptom score [TNSS]), the secondary evaluation outcome was the Rhinoconjunctivitis Quality of Life Questionnaire (RQLQ) and adverse events. The Cochrane Collaboration's Tool was used to evaluate the methodological quality of articles, the Grading of Recommendations Assessment, Development and Evaluation (GRADE) instrument was used to evaluate the quality of evidence. Network meta-analysis was completed by using Stata Statistical Software (Version 14.0, Stata Corporation, College Station, TX).

**Results:**

This study will compare and rank the different traditional Chinese medicine therapies for allergic rhinitis.

**Conclusion:**

This study is the first time to use network meta-analysis (NMA) to compare the efficacy and safety of traditional Chinese medicine for the treatment of allergic rhinitis, which will provide ideas and methods for the clinical treatment for allergic rhinitis.

**INPLASY registration number:**

No. INPLASY202080119

## 1 Introduction

Allergic rhinitis (AR) is a common nasal mucosal noninfectious inflammatory disease, mainly caused by the pathogenesis of atopy individuals after exposure to allergens, mediated by immunoglobulin E (IgE) media release, then to stimulate the nasal mucosa sensory nerve endings and blood vessels, and to exhilarate sympathetic nerve. The clinical symptoms were usually characterized by nasal itching, nasal congestion, sneezing, runny nose, if the symptoms were not released promptly, it will also cause other symptoms such as headache, dizziness.^[1–3]^ In addition, studies have shown that allergic rhinitis is associated with asthma, sinusitis, conjunctivitis, and chronic cough.^[3,4–6]^

Although allergic rhinitis does not cause death in humans, it seriously affects people's daily life and work.^[7,8]^ According to the epidemiological survey of global prevalence, the incidence of allergic rhinitis is about 10% to 20% globally,^[9]^ due to the influence of the environments, the prevalence of various regions have differences, the prevalence in United States is 10% to 30%,^[10]^ in Sweden it is about 28%,^[11]^ in mainland China it is 4% to 38%,^[12]^ and studies showed that the prevalence of allergic rhinitis are trending up.^[13,14]^

So far, the treatment of allergic rhinitis is mainly by avoiding exposure to allergens and drug therapy.^[15]^ But, as we have seen, it is impossible to be exposed completely to allergens. On the other hand, western drugs are mainly the corticosteroids and antihistamines, taking these drugs in long-term will cause side effects such as obesity, arrhythmia, and dry mouth, which will affect human health.^[16]^

Considering these factors, current to search a treatment that is non-toxic and has no side effects is what we should focus on. Traditional Chinese medicine therapy for allergic rhinitis has achieved good results, and no harm to human body for the time being.^[17–19]^ However, TCM therapy includes many types: acupuncture, moxibustion, acupoint sticking, etc., but we don’t know yet which therapeutic measure is more effective. Therefore, it is necessary to evaluate the efficacy and safety of different TCM therapies for allergic rhinitis.

## 2 Methods and analyses

### 2.1 Design

Systematic review and network meta-analysis.

### 2.2 Patient and public involvement

This study is a secondary literature study and does not involve clinical patients or the general public.

### 2.3 Eligibility criteria

#### 2.3.1 Types of studies

Considering that the methodological quality of this paper is crucial to the conclusion, we only included the randomized controlled trials (RCTs) of traditional Chinese medicine therapy for allergic rhinitis, and the traditional Chinese medicine therapy only limited to acupuncture, moxibustion, and acupoint sticking, while traditional Chinese medicine and massage were excluded. Studies of one TCM therapy versus another TCM therapy or placebo were analyzed.

#### 2.3.2 Type of participant

Among all adults (over 18 years old) were diagnosed with allergic rhinitis. The diagnostic criteria were derived from ARIA (Rhinitis and its Impact on Asthma) Guidelines,^[20]^ The Rhinitis - Clinical Investigator Collaborative (AR-CIC),^[21]^ Chinese Journal of Otorhinolyngology Head and Neck Surgery.^[22]^

#### 2.3.3 Interventions

The intervention measures are traditional Chinese medicine (limited to acupuncture, moxibustion, and acupoint application) for the treatment of allergic rhinitis. Among them, the acupuncture includes electroacupuncture, fire acupuncture, plum flower acupuncture, etc., the moxibustion includes ginger moxibustion, suspended moxibustion, fester moxibustion, etc. There are no restrictions on the traditional Chinese medicine used in acupoint application.

#### 2.3.4 Types of outcome measurements

##### 2.3.4.1 Primary outcome

The total nasal symptom score (TNSS)^[23]^ was applied for scoring nasal congestion, nasal mucus, rhinitis, and sneezing with a total of 12 points. The higher score means the more severed of the symptoms.

##### 2.3.4.2 Secondary outcomes

(1) The Rhinoconjunctivitis Quality of Life Questionnaire (RQLQ),^[24]^ the patients’ quality of life were evaluated from 28 questions in 7 dimensions: activity, sleep, non-nasal and eye symptoms, nasal symptoms, eye symptoms, emotion and practical problems, with a total score of 42 points. The higher score means the worse of the life quality.
(2) The incidence rate of adverse events.

#### 2.3.5 Exclusion criteria

(1) Exclusion of reviews, animal experiments, case reports, and non-randomized controlled trials.
(2) Exclusion of comparison to the treatment of different acupuncture techniques or moxibustion methods.
(3) If there is any information lost or obvious error in the article, it will be screened out for further study.

### 2.4 Literature search

Comprehensive searches of RCTS on traditional Chinese medicine therapy for allergic rhinitis were conducted in 3 English databases of PubMed, Cochrane Library, Embase, and 2 Chinese databases of CNKI and Wanfang, and the time of index was from their inceptions to September 1, 2020 for each database. The retrieval strategy of PubMed is shown in Table 1. Two team members (YT and YSF) independently searched the article according to the retrieval strategy, they also exported the citations.

**Table 1:**
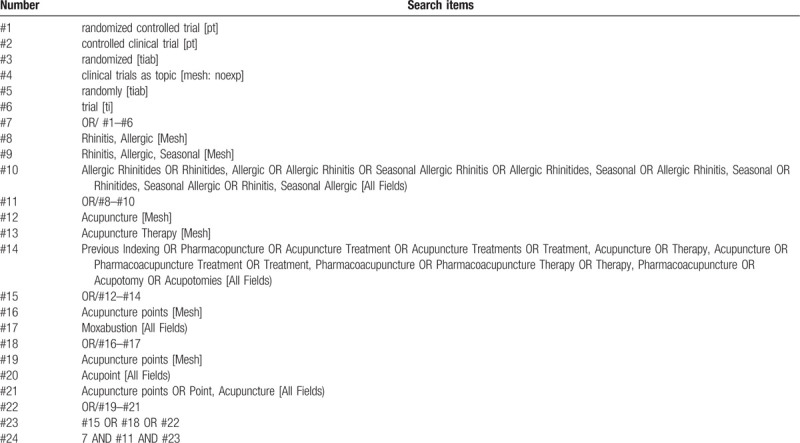
Search strategy used in PubMed database.

### 2.5 Data collection

#### 2.5.1 Selection of studies

**Figure 1:**
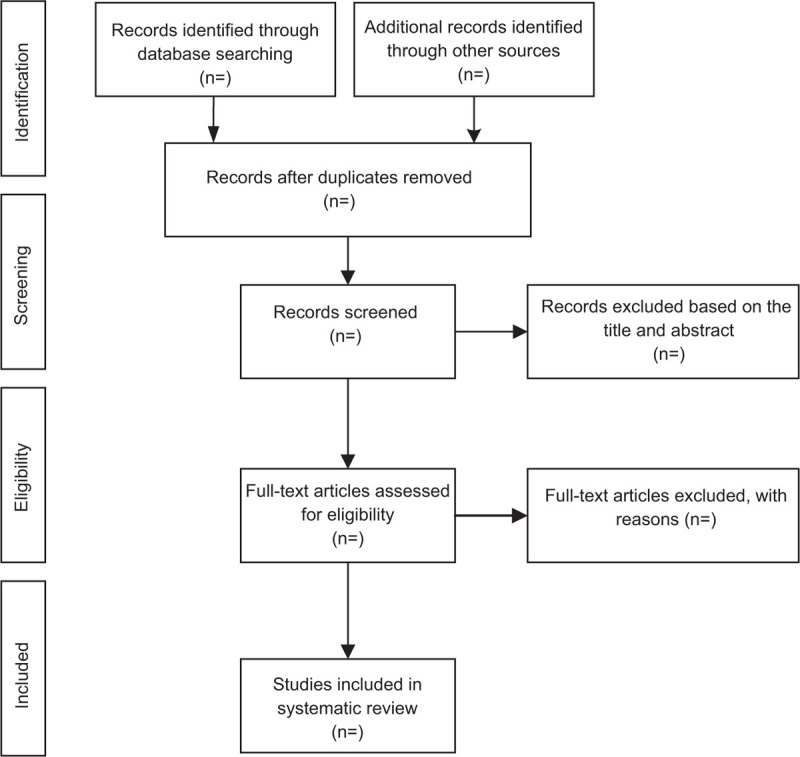
Flow diagram of study selection process.

For the convenience of management, we searched from 5 databases and imported titles into EndNote Software AQ8 (V.X9). Firstly, we used the software to remove duplicate articles, then 2 team members (YT and YSF) independently read the titles and abstractions, they deleted the literature that did not meet the requirements, and read the full text of the remained articles to decide the final inclusion of the experiment. After that, cross-checking to the results of both parties was conducted. If there is any disagreement, the decision would be made via group discussion. The entire process and results are shown in Fig. 1.

#### 2.5.2 Data extraction and management

Microsoft Excel 2016 was used to establish information data extraction table, and pre-extraction was carried out to determine the feasibility of the table. Then 2 team members (YT and YSF) independently extracted the following information after training:

(1) Basic information: title, author, country, year, language, etc.
(2) Baseline information: gender, age, number of persons, country, diagnostic criteria, etc.
(3) Methodological information: grouping method, allocation concealment, blind method, result bias, etc.
(4) Intervention measures: treatment measures, treatment time, frequency, etc.
(5) Results: data of primary and secondary results.

After the work is completed, the results are cross-checked, if there are differences, a group discussion is conducted to determine the final result.

### 2.6 Assessment of risk of bias in included studies

The 2 authors (YT and YSF) evaluated the article methodology of inclusive trials independently, by the Cochrane collaboration “Bias risk” tool sequences generated from 6 aspects of allocation concealment, blind (or mask), incomplete data evaluation, evaluation reports, and other sources of bias selective results. Finally, for each items, we made ranking of “Low-risk bias,” “High-risk bias,” and “Unclear” based on the Cochrane collaboration “bias risk” tool.^[25,26]^

### 2.7 Data analysis

#### 2.7.1 Management of lost data

If data are insufficient from the selected study, we will contact the author via email for the required data. If baseline and outcome data or other data are included, the mean and standard deviation of the change will be manually calculated according to the Cochrane.^[27]^

#### 2.7.2 Network map

In the network diagram, each dot represents an intervention; the larger dot area means the bigger population of the studied intervention; the line between the 2 dots represents that there is direct comparison to RCT studies among 2 interventions; the line thickness represents the numbers of direct comparison to RCT studies among 2 interventions.

#### 2.7.3 Transitivity and consistency assessment

Transitivity and consistency are the prerequisites for reticular meta-analysis. The transitivity was evaluated qualitatively from the perspective of methodology and was evaluated according to the PICO principle. Consistency was mainly to check local and overall consistency. Local consistency can be checked by loop consistency test (Higgins model). The global consistency test was verified by the corresponding inconsistency model according to different data.

#### 2.7.4 Assessment of heterogeneity

Heterogeneity tests for all included studies were performed by using Network prediction interval graph, then to study the relationship of the weighted mean difference (WMD) at a 95% confidence interval (95% CI) and estimation zone (95%Prl) to invalid line, only when invalid line crosses perpendicularly to estimation zone but doesn’t to confidence interval, then means heterogeneity exists.^[28]^

#### 2.7.5 Pairwise meta-analysis

If there is a direct comparison between the experimental interventions included in the data (TCM vs TCM, TCM vs placebo), the Stata14.0 will be used for pairwise meta-analysis based on a random-effects model.

#### 2.7.6 Network meta-analysis

Two team members (YT and YSF) used statistical software - Stata (version 14.0, Stata Corporation, College Station, TX) for analysis. A random effects model was used for network meta-analysis to compare the variables between different interventions. By comparing surface under the cumulative ranking curve (SUCRA), the optimum intervention measures were determined. The range of SUCRA is 0% to 100%, the higher of the cumulative ranking curve means the better of the efficacy.^[29]^

#### 2.7.7 Assessment of reporting biases

Funnel plots are used to detect publication bias. If the images are asymmetric, it indicates that there is publication bias.

#### 2.7.8 Subgroup analysis

If the analysis shows significant heterogeneity, then the root cause will be analyzed according to the PICOS principle, and the STATA 14.0 will be used for subgroup analysis.

#### 2.7.9 Grading the quality of evidence

According to the standards in the Grading of Recommendations Assessment Development and Evaluation (GRADE) system,^[30]^ 2 team members evaluate the quality of the research and divide it into 4 levels of “high,” “medium,” “low,” and “very low,” then the results will be exchanged. If there is any disagreement, the final option will be selected via group discussion.

### 2.8 Ethics and dissemination

The secondary literature study has no relationship to the personal data of the study, so the ethical approval is not required. Evaluation of the efficacy and safety of different traditional Chinese medicine therapies for allergic rhinitis may provide evidence for clinical treatment of this disease. The results of the study will be published in a peer-reviewed journal.

## Author contributions

**Data curation:** Ting Yu, ShiFan Yan.

**Formal analysis:** Ting Yu, ShiFan Yan, HaiYan Li.

**Funding acquisition:** Lin Jiao.

**Investigation:** SiYu Qin.

**Methodology:** Ting Yu, ShiFan Yan, MingHui Li.

**Project administration:** Rixin Chen.

**Resources:** HaiYan Li, SiYu Qin, MingHui Li.

**Software:** ZhenHai Chi, Pan Cheng.

**Supervision:** ZhenHai Chi, Pan Cheng.

**Writing – original draft:** Lin Jiao.

**Writing – review & editing:** Rixin Chen.
